# Supplementary figures and images for: Agricultural big data and methods and models for food security analysis—a mini-review
Source: PeerJ. 2022 Jun 29;10:e13674. doi: 10.7717/peerj.13674 (PMC9250308; doi:10.7717/peerj.13674)

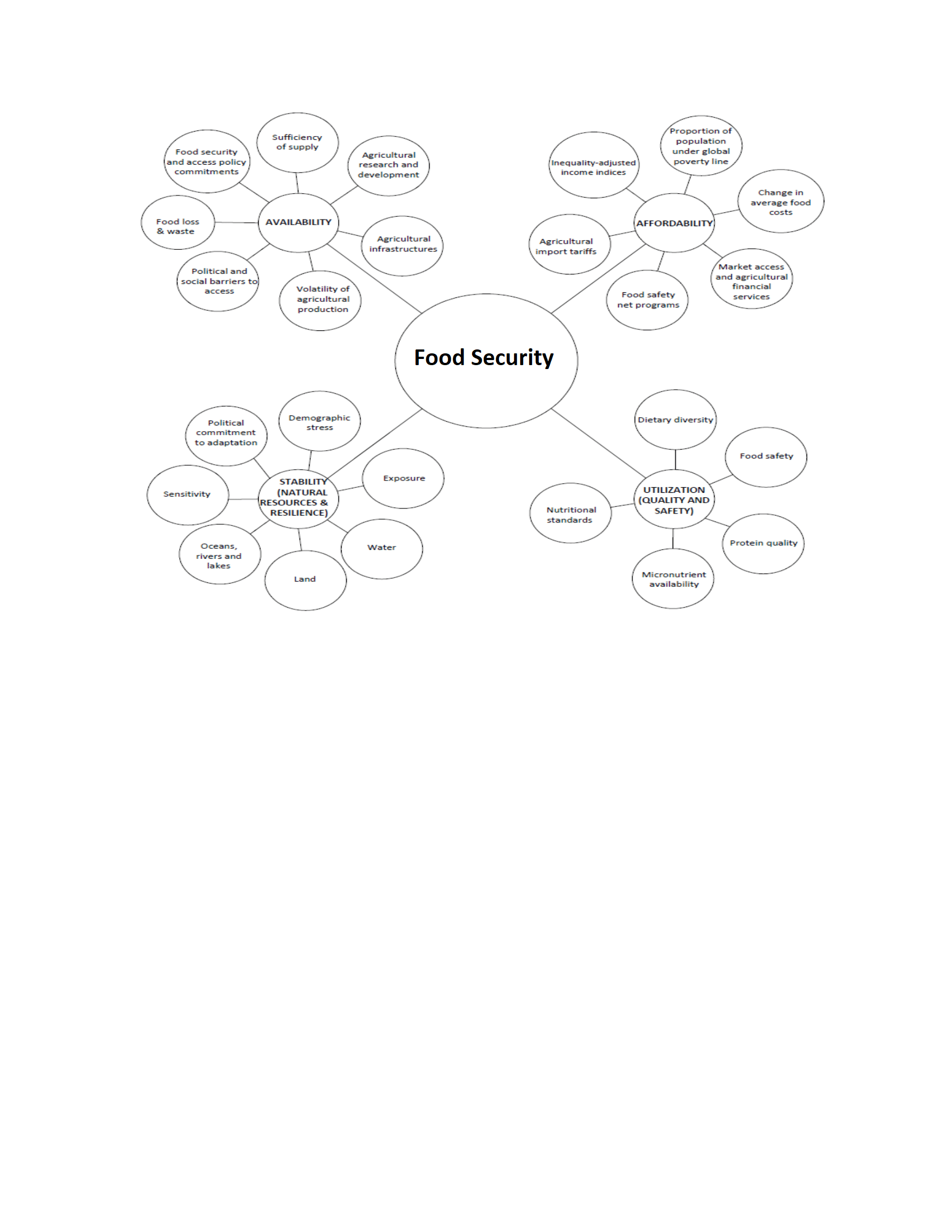

Supplement: Figure S1 — Data source: FAO. 2019. The State of Food and Agriculture 2019. Moving forward on food loss and waste reduction. Rome. Licence: CC BY-NC-SA 3.0 IGO. [file peerj-10-13674-s001.png]
