# Supplementary material for: Agricultural big data and methods and models for food security analysis—a mini-review
Source: PeerJ. 2022 Jun 29;10:e13674. doi: 10.7717/peerj.13674 (PMC9250308; doi:10.7717/peerj.13674)
Supplement: Table S1 [file peerj-10-13674-s002.docx]

S. Table 1. Reviewed food security models based on FS dimensions

| No. | Reference | Classification | Calculations of Food Security dimensions | | | | |
| --- | --- | --- | --- | --- | --- | --- | --- |
|  |  |  | Availability | Access | Utilization | Stability | Others |
| 1 | (Adewumi and Animashaun, 2013) | Statistical |  | production efficiency and HDDS correlation |  |  |  |
| 2 | (Ahmad et al., 2016) | Statistical |  |  |  |  | FSI developed by PCA analysis of different variables include per capita consumption |
| 3 | (Akerele and Shittu, 2017) | Statistical |  | Based on household survey data, Berry and Relative Entropy Index values for dietary diversity using seven food groups, regressed on food prices and household characteristics. |  |  |  |
| 4 | (Akerele et al., 2017) | Statistical |  | Based on household survey data, Berry and Relative Entropy Index values for dietary diversity using seven food groups, regressed on food prices and household characteristics. |  |  |  |
| 5 | (Akinola et al., 2010) | Statistical |  | Crop yields are used to estimate food consumption in terms of expenditures, calories, and protein. |  |  |  |
| 6 | (Ali and Erenstein, 2017) | Statistical |  | To determine food security, food consumption is compared to a consumption threshold. |  |  |  |
| 7 | (Alwang and Siegel, 1999) | Optimization |  | Food consumption is a constraint in an optimization model |  |  |  |
| 8 | (Amede and Delve, 2008) | Optimization | Food production is a constraint in an optimization model |  |  |  |  |
| 9 | (Azeem et al., 2016) | Statistical |  | Household, locational, and community characteristics, as well as their cross-terms, are regressed on calorie intake in relation to a recommended value. |  |  |  |
| 10 | (Bacon et al., 2014) | Statistical |  | Crop production, land area, number of livesotck, household size, farming practices, location community involvement, and coping mechanisms are all regressed on the number of 'lean months.' |  |  |  |
| 11 | (Baran et al., 2010) | Statistical |  |  |  |  | Bayesian Belief Networks are used in the analysis, with probabilities estimated by stakeholders. Probabilities are for "good" or "bad" outcomes for production, income, and an undefined "food security" outcome. Alternative water management strategies' effects are described in terms of the likelihood of good or bad outcomes. |
| 12 | (Bashir et al., 2014) | Statistical |  | 2SLS is used to regress calorie intake on household characteristics such as income, livestock assets, education, and the age of the household head. |  |  |  |
| 13 | (Beghin and Teshome, 2017) | Statistical |  | The "food shortage" scale is used to divide the sample into food secure and food insecure groups, and differences in characteristics are statistically tested. Logit models use land area, number of children, off-farm labor, and the sex of the household head to assess food security. |  |  |  |
| 14 | (Beyene and Muche, 2010) | CGE |  | With independent variables such as farming activities, source of income, land and livestock holdings, input use, and soil and water conservation practices, a logit model is used to assess calorie acquisition. |  |  |  |
| 15 | (Bharwani et al., 2005) | Simulation biophysical | Water availability and evapo-transpiration drive crop yields in this agent-based model. |  |  |  |  |
| 16 | (Beyene and Engida, 2016) | CGE | The impact of farmer training and irrigation on agricultural production was assessed using the CGE model. Production is modeled as a function of labor and capital, with capital including land, livestock, and other non-agricultural assets. |  |  |  |  |
| 17 | (Darsonon., 2017) | Statistical | Own rice production indicates food availability, and food insecure households produce less than a certain amount of rice. |  |  |  |  |
| 18 | (Dhakal et al., 2010) | Optimization | With implications for food production, land allocation is modeled to maximize community income. The yields per land area times area model is used to calculate food production. |  |  |  |  |
| 19 | (Di Falco et al., 2011) | Statistical | A household head and farm charateristics-based endogenous switching regression model was used to investigate the effects on grain yield per hectare. |  |  |  |  |
| 20 | (Dil Farzana et al., 2017) | Statistical |  | The HFIAS scores were used to identify which households had "low" food security, and their coping strategies were then described. |  |  |  |
| 21 | (Djanibekov et al., 2013) | Optimization |  | To determine food consumption, the model includes a quadratic function of household income. |  |  |  |
| 22 | (Sohoulande Djebou et al., 2017) | Statistical |  | A statistical model with land size, irrigation, fertilizer, and tractor use as explanatory variables uses a food access indicator similar to FIES. |  |  |  |
| 23 | (Dobbie. et al., 2017) | Simulation integrated | Food production decisions and thus caloric availability are determined by an agent-based model. | The food accessed and dietary diversity are determined by the agent-based model (food counts) |  |  |  |
| 24 | (Ferdous et al., 2016) | Other | The effectiveness of a home garden intervention is assessed. |  |  |  |  |
| 25 | (Gangwar et al., 2016) | Simulation biophysical |  |  |  |  | Based on farm-level implementation results, a set of production practices ("Farming system model") was "synthesized" and used to describe the impacts on production and net income. The percentage of total requirements for "balanced nutrition" is given, but no further information is given. |
| 26 | (Habyarimana and Nkunzimana, 2017) | Statistical |  | The effect of household expenditure, crop yields, and access to extension services on the Food Consumption Score is quantified using pooled OLS. |  |  |  |
| 27 | (Hadush, 2017) | Statistical |  | The effects of household production and characteristics on per capita food consumption expenditure are quantified using a 2SLS model. |  |  |  |
| 28 | (Hammond et al., 2018) | Others | Food availability is a supply-based estimate of the amount of food that can be generated by any one household through on- and off-farm activities, measured in kilocalories (kCal) per person (male adult equivalent) per day. | HFIES and HDDS |  |  |  |
| 29 | (Hartter and Boston, 2007) | Simulation integrated | With fixed coefficients per activity, production decisions are driven by per capita caloric needs. | Per capita caloric needs drive production decisions, with fixed coefficients per activity. |  |  |  |
| 30 | (Hoddinott et al., 2012) | Statistical | Dose-response models assess the impact of transfer program participation on yields and production. |  |  |  |  |
| 31 | (Holden and Shiferaw, 2004) | Optimization | The soil type, soil depth, slope, fertilizer application, and manure application all affect crop yields. Livestock productivity, birth rates, mortality, feed requirements, milk production, ploughing capacity, manure production, culling rates, and other input costs are all considered. | Based on consumption data, an extended quadratic expenditure system was estimated, which included food grain, pulse, and other consumption, as well as farm input expenditure. |  |  |  |
| 32 | (Holden et al., 2005) | Optimization | Same to No 31 | Same to No 31 |  |  |  |
| 33 | (Hussien et al., 2017) | Simulation integrated |  | The amount of food consumed by a household is a simple function of its size and income. |  |  |  |
| 34 | (Ibrahim et al., 2009) | Optimization | Food security is a caloric production constraint in an optimization model for farm production. |  |  |  |  |
| 35 | (Inder et al., 2017) | Statistical | Crop yields are predicted using median regression models as a function of land area, tree density, labor availability, and location. | Land area, household labor availability, type of roofing, household head education, and location are all regressed on ad hoc food security indicators (food shortages experienced and months of food shortages). |  |  |  |
| 36 | (Islam et al., 2018) | Statistical |  | The relationship between farm diversification and diet diversity (HDDS, Women's Dietary Diversity Score, WDDS, and the Food Variety Score, FVS) was investigated in this study. The total number of crop, vegetable, and fruit species, the number of food crop species only, and the Margalef species richness index were used in farm diversification. |  |  |  |
| 37 | (Joshi and Joshi, 2017) | Statistical |  |  |  |  | Explanatory variables include the age of the household head, household size, irrigation use, distance to market, livestock, land ownership, and district in a logistic regression model with a binary variable for "food security" (not clearly defined). |
| 38 | (Nyaga and Doppler, 2009) | Statistical |  |  |  |  | Using variables representing production, farming assets, and land area and location, PCA is used to create a "food security index." In a logistic regression with household and farm characteristics, this PCA index is used. There is no economic or other logic behind the variables chosen. |
| 39 | (Kaminski and Thomas, 2011) | Optimization |  |  |  |  | An unspecified "food security goal" is included in an ordered discrete choice model of cotton production decisions. |
| 40 | (Karki et al., 2015) | Statistical |  |  |  |  | Used classification and regression trees (CART) to highlight the primary associations between dependent variables of interest (food self- sufficiency, crop yield and adoption of improved cultivars) and independent variables belonging to the five subject areas included in the survey (socioeconomic, land tenure, labor, crop production and animal husbandry). |
| 41 | (Kassie et al., 2015) | Statistical |  |  |  |  | A variety of ad hoc food security indicators reported by households was regressed on variable for human capital, resource constraints, market access, social capital, experience of shocks and natural capital. |
| 42 | (Kassie et al., 2008) | Statistical | **value of crop production per hectare** in low and high rainfall areas of the Ethiopian highlands using cross-sectional data from more than 900 households, with multiple plots per household |  |  |  |  |
| 43 | (Kokoye et al., 2013) | Optimization | A linear programming model to maximize income subject to constraints on land, labor, credit, seed, and the production required to meet household consumption needs (from own production), which is equated with food security. |  |  |  |  |
| 44 | (Kowero et al., 2005) | Optimization | Optimization model (weighted goal programming) with constraints on land and labor and the production of crops and forestry products. |  |  |  |  |
| 45 | (Laborte et al., 2007) | Optimization | Optimization models of agricultural production and income at three levels of aggregation: farm, municipality and province. The model results focus on yields, areas, gross income, net income and N losses. **Food availability** is represented by yields and production, but is not explicitly linked to food security other than to mention that this is a goal at the municipality and provincial levels. |  |  |  |  |
| 46 | (Laborte et al., 2009) | Optimization | Optimization model of the farm to assess impacts of addopting agricultural technologies. The model results focus on yields, areas, gross income, net income and N losses. **Food availability** is represented by yields and production. |  |  |  |  |
| 47 | (Lázár et al., 2015) | Simulation integrated | CROPWAT model is used to generate yields and production, which are valued at market prices. The net income from farming is used to approximate alllowable expenditures on calories, and the number of months where calories are below minimum recommended thresholds is calculated as "hunger periods |  |  |  |  |
| 48 | (Leonardo et al., 2018) | Optimization | A linear programming model to maximize gross margin or maize sales subject to resource and production constraints and amounts required to meet household caloric needs (from own production), which is equated with food security. |  |  |  |  |
| 49 | (Louhichi and Gomez y Paloma, 2014) | Optimization | A non-linear optimization model which includes the household’s utility framework and the farm’s production constraints, in a non-separable regime.  Production and consumption amounts are reported as changes from baseline | A Linear Expenditure System (LES) represents household consumption decisions, and production and consumption amounts are reported as changes from baseline |  |  |  |
| 50 | (Maatman et al., 1998) | Optimization | A stochastic linear programming model to assess the impact of rainfall risks food surplus or deficit based on of energy requirements. **Food availability** is modeled as food production.  Food consumption is represented as requirements, with sales or purchases based on the adequacy of production. |  |  |  |  |
| 51 | (Magcale-Macandog et al., 2010) | Simulation biophysical | The Water, Nutrient and Light Capture in Agroforestry Systems (WaNuLCAS) model was used to determine the complementary effects of trees and crops in the agroforestry systems in improving crop yields. WaNuLCAS scenarios indicate income from production and amount of cash available for the households that could be used to purchase food. |  |  |  |  |
| 52 | (Marsh et al., 2016) | Statistical |  | A two-stage linear regression model explains household food expenditure. **Food consumption** is represented as aggregated food expenditures and is explained by independent variables: six binary income category variables, household size and predicted number of vaccinated cattle. |  |  |  |
| 53 | (Modi, 2015) | Statistical |  |  |  |  | "Food security value" is measured with a equation including yields (availability), home consumption (access) and locally marketed crop (marketability), all expressed in terms of tons/ha. This calclulation is applied separately to eight crops (cabbabe, swiss chard, maize, beans, sweet pottaoes, taro, and potatoes). |
| 54 | (Molua, 2012) | Statistical |  | A multinomial logit model is used to explain changes in household food consumption patterns, farming practices and market orientation, with the independent variables: household size, farm size, education, wealth, credit access, land tenure, climate variation and extension service. |  |  |  |
| 55 | (Murungweni et al., 2011) | Simulation integrated |  |  |  |  | Fuzzy Cognitive Maps (FCM) developed with stakeholders are link variables using numerical "influences" among variables. Food in household is positively influenced by "total harvest", "garden crop harvest", and "cash", which is in turn positively ifluenced by "cattle sold". No specific quantified food security indcators are provided. |
| 56 | (N’Danikou et al., 2017) | Statistical |  | Mixed effects (recursive partitioning tree) models fitted by maximum likelihood were used to examine determinants of a "food insecurity index" defined as the weighted average score of the severity of a Coping Strategy Index (CSI).  Explanatory variables used a livelihoods categorization: human capital=risk attidude, natural capital=richness of wild food plants, financial capital=income from non-ag employment, social capital=social group membership and responsibilities in the community. |  |  |  |
| 57 | (Niragira et al., 2015) | Optimization | A mathematical programming to obtain the optimal crop mix that could maximize output given the constraints on production factor endowments and the need to feed the household. The objective function and constraints are not completely described. Some model scenarios include household consumption requirements for energy, protein and fatty acids. **Food availabilty** is represented by own production. |  |  |  |  |
| 58 | (Nkegbe et al., 2017) | Statistical |  | A probility model with three categories (little or no hunger, moderate hunger, severe hunger) to examine determinants of the Household Hunger Scale (HHS) as a measure of **food access**.  Independent variables include age, gender, education and marital status of the household head, household size, ownership of transport, equipment, yield index for maize, binary variables for ownership of land and animal assets, food consumption expenditures (FCE), and region. Note that FCE are used here as an explanatory variable (technically, endogenous) for HHS. |  |  |  |
| 59 | (Obayelu and Onasanya, 2016) | Statistical |  | Food security is represented with an index that divides average self-reported daily per capita caloric intake for each household by recommeded caloric intake. Logit regression models (OLS and 2SLS) to explain caloric consumption (**food access**) include independent variables: quantity produced, age, gender and education of household head, houshold size, number of dependents, binary variable for cooperative membership, years farming experience, farm size, "food security status" ("secure" or "insecure"), extension contact, farm income, non-farm income, credit access, occupation. |  |  |  |
| 60 | (Ogot et al., 2017) | Statistical | This study compared several outcomes between farm households using or not using a Push-Pull Technology (PPT) for insect control. **Food availability** includes crop production.  These are comparisons of means without statistical tests for significance or controlling for other factors. There is no empirical or explanatory model analysis. | This study compared several outcomes between farm households using or not using a Push-Pull Technology (PPT) for insect control. **Food access** is measured by "food shortage" by month, food expenditures, the Household Dietary Diversity Score (HDDS). These are comparisons of means without statistical tests for significance or controlling for other factors. There is no empirical or explanatory model analysis. | This study compared a number of outcomes between farm households using or not using a Push-Pull Technology (PPT) for insect control. **Food utilization** is measured by BMI scores for < 5 years and > 5 years. These are comparisons of means without statistical tests for significance or controlling for other factors.  There is no empirical or explanatory model analysis. |  |  |
| 61 | (Jiang et al., 2012) | Statistical | This study uses a random effects model based on panel data to assess factors influencing grain production per capita. Observational units are not clearly defined, but explanatory variables include: altitude, soil organic matter, temperature, hours of sunshine, road network density, "investment", but rather curiously also yields, production and population |  |  |  |  |
| 62 | (Radchenko and Corral, 2018) | Statistical |  | This study uses a semi-parametric approach to examine the impact of cash cropping on Food Consumption Score (FCS) and food consumption expenditures, both indicators of **food access**. A probit model predicts the likelihood of a household engaging in cash cropping and the semi-parametric model uses independent variables: aggregated value of agricultural production age, sex and education of household head, household size, child dependency ratio, distance to populaiton center, border and tobacco auction, binary variables for district, and access to manufactured goods and access to staple foods reported as "stable". |  |  |  |
| 63 | (Rader et al., 2009) | Optimization | A stochastic mixed integer programming decision support system (DSS) that determines optimal crop planting practices to minimize the caloric deficit of a household and maximize limited household income given a seasonal probabilistic rainfall forecast and the farmer’s risk level. The objective function minimizes the expected value of the caloric deficit minus the expected cash assets multiplied by a small number. The definition of caloric deficit implicitly equates own consumption with production, thus conflating measures of **food availability** and **food access**. The caloric deficit assumes standard caloric values per kg of food produced, and is calculated relative to an optimal consumption level. |  |  |  |  |
| 64 | (Ragasa and Mazunda, 2018) | Statistical | This study formulates three econometric models (Correlated Random Effects, CRE; Instrumental Variables, IV; and Control Function, CF) to assess the determinants of yields and food security. **Food availability** is represented by the LN of the value of production per hectare (not quantity). Independent variables explaining food access are: quantity of subsidized and unsubsidized fertilizer per ha, receipt of extension advice, use of varieties and technologies, number of plots, land owned, asset value index, access to credit, household size, age, education and sex of household head, rainfall, distance to nearest paved road and marketing outlet, and binary variable for whether community has a health facility | This study formulates three econometric models (Correlated Random Effects, CRE; Instrumental Variables, IV; and Control Function, CF) to assess the determinants of yields and food security. **Food access** indicators include Household Dietary Diversity Score (HDDS) and Food Consumption Score (FCS).  Independent variables explaining food access are: quantity of subsidized and unsubsidized fertilizer per ha, receipt of extension advice, use of varieties and technologies, number of plots, land owned, asset value index, access to credit, household size, age, education and sex of household head, rainfall, distance to nearest paved road and marketing outlet, and binary variable for whether community has a health facility. |  |  |  |
| 65 | (Reincke et al., 2018) | Statistical | This study uses regression analysis to assess the determinants of food availability and food access. **Food availability** is measured with a synthetic index based on qualitative indicators harvest quality and premature harvesting of crops. Each of these indicators is regressed on a set of independent variables including number of mechanical assets, cultivation of individual crops, participation in collective agriculture, affected by high food prices, drought or pests, value of inputs purchases, number of household members, receipt of food aid, access to information about food conservatoin and processing, total income, literacy, share of infertile land, wage employment. Many of these variables could be considered endogenous and the authors do not modify regression methods to account for this. | This study uses regression analysis to assess the determinants of food availability and food access. **Food access** was measured with the HFIAS and a dietary diversity index (DDI, which the authors consider a measure of utilization). Each of these indicators is regressed on a set of independent variables including number of mechanical assets, cultivation of individual crops, participation in collective agriculture, affected by high food prices, drought or pests, value of inputs purchases, number of household members, receipt of food aid, access to information about food conservatoin and processing, total income, literacy, share of infertile land, wage employment. Many of these variables could be considered endogenous and the authors do not modify regression methods to account for this. |  |  |  |
| 66 | (Rigolot et al., 2017) | Simulation integrated | **Food availability** is defined as total caloric energy produced per year, and is simulated with the combination of the APSIM and LivSIM models. (The authors do not indicate how the energy content of production was determined.) |  |  |  |  |
| 67 | (Salazar et al., 2016) | Statistical | **Food availability** is (indirectly) measured as the value of production per ha and total and is analyzed with the same econometric approach as for food access. | **Food access** is measured by the ELCSA experiential scale. Two stages of econometric model predict participation in a program designed to increase agricultural productivity with indpendent variables household size, dependency ratio, education, type of housing, cooperative membership, proportion agricultural income, credit access, remittance income, livestock owned, land ownership, distance to paved road and program sites. The impact of program participation on food security uses the likelihood or program participation on the overeall index value and individual questions from the ELCSA (rather than just the overall index value as is more typical) for all households and those with chidren under 18 years. Food access regressions have many of the independent variables identified above. |  |  |  |
| 68 | (Sassi and Cardaci, 2013) | CGE | **Food availability** is measured as aggregrate production, with a focus on the yield impacts of rainfall, which are estimated with simple linear regression models including a trend term. This is dervied from scenario analysis with a Computable General Equilibrium (CGE) model. | **Food access** is measured as food consumption expenditures. This is dervied from scenario analysis with a Computable General Equilibrium (CGE) model. |  |  |  |
| 69 | (Seaman et al., 2014) | Simulation, other |  |  |  |  | Household "food access" is described by an equation that converts changes in the value of production into caloric equivalents and compares it to a household food requirement. The notation used for this equation is convoluted and it is difficult to assess whether the conversion of units of measure (e.g., money to kcal) are consistent. |
| 70 | (Sibhatu et al., 2015) | Statistical |  | **Food access** is measured by HDDS based on 10 and 12 food groups, consumption of fruits and vegetables, caloric intake, and intakes of iron, zinc and Vitamin A. This is analyzed with a regression model that includes a measure of farm production diversity based on the same food groups, and land area cultivated, share of land in food crops, cash revenues from agriculture, total household income, distance to market, household size, age education and sex of the household head. |  |  |  |
| 71 | (Stephens et al., 2012) | Simulation integrated | The study used a system dynamics model to assess the complex interactions of biophysical and socioeconomic factors and their feedback with household economic decision making. The CLASSES model, models the key inflow, outflow and feedback processes that determine levels of biophysical and economic stock variables  at each point in time. The biophysical sub- models produce estimates of yield, as indicators of **food availability**. | The socio-economic section of the model simulates consumption (a food access indicator), based on the availability of grain, livestock, and cash. |  |  |  |
| 72 | (Suneetha and Yirgu, 2010) | Statistical | This study examined the statistical relationship between independent household variables and household **food availability. Food availability** was measured as calories available per capita based on production, purchases, sales, food-for- work, other receipts and post harvest losses, as "food balance sheet approach" often used as more aggregated levels. A single-equation linear regression model assessed the impacts of characteristics of the household (farm size, livestock ownership, input access and use, and the age, education and sex of the household head. |  |  |  |  |
| 73 | (Szabo et al., 2016) | Statistical |  |  |  |  | A logit regression models to assess factors affecting the proportion of household expenditure on food and the caloric availability. Food security is defined expenditures on food less than 75% of the total and caloric availability (intake, they appear to be used interchangeably) is above "requirements". Explanatory variables include salinity, wealth quintile, religion, household head characteristics, agricultural activities of household and remittance income. |
| 74 | (Tesfaye et al., 2008) | Statistical |  | The study assessed the effect of an irrigation scheme on household food security. **Food access** data (consumption) were collected through a random sampling and interview process.  Statistical methods (the Heckman two-step procedure) used independent variables: household reesource endowments, household characteristics and access to information and services |  |  |  |
| 75 | (Thorlakson and Neufeldt, 2012) | Statistical | A linear regression on matched data for households using and not using agroforestry to assess the impact of the value of production, which is a measure of **food availability**. The independent variables are not specified, but included "household parameters".  No specific food security impacts were analyzed |  |  |  | The study indicates that farmers define food security as: "ability to obtain an adequate diet for allhousehold members throughout the year, without beingforced to use long-term savings to purchase food". This measure was not assessed in the analysis. |
| 76 | (Thornton et al., 2006) | Simulation integrated | The Savanna model spatially models interactions between livestock and wildlife. Joined to Savanna is a household model (PHEWS) which tracks resources, cash and dietary energy. **Food availability** is assessed by calculating the energy requirements of the household, specifying rules for the make-up of diets, and determining outcomes based on resources and cash. |  |  |  |  |
| 77 | (Tingem et al., 2008) | Simulation biophysical | This study combines a weather generation simulator (ClimGen) with a crop growth model (CropSys), and assesses whether prediction of wet/dry years can improve household outcomes. The results are assessed in terms of variability in crop yields, which are used as indicators of **food availability**. |  |  |  |  |
| 78 | (Tittonell et al., 2009) | Simulation biophysical | The FarmSIM model was used to evaluate case study farms with different resource endowments. FarmSIM is a dynamic model that links different household and biophysical sub-models at the farm scale. Different crop- livestock integration scenarios were assessed, with the model simulating crop and livestock production, sales and consumption, and the capacity to meet the energy requirements of the household through on-farm food production |  |  |  |  |
| 79 | (Traore et al., 2017) | Simulation biophysical | The APSIM crop model was used to simulate climate risks for cereal crops, based on climate change scenarios. Crop yield was the main output, and results were scaled for three different farm types with differing resources. **Food availability** was defined as self sufficiency according to the balance of farm-level crop production and requirements. |  |  |  |  |
| 80 | (Traoré et al., 2018) | Statistical |  | This study used linear mixed models with village and commune random effects to examine the relationship between different cattle breeds , household wealth, dependency ratio, education, cotton cultivation and location on f**ood acces**s measured with HDDS, FCS and mHFIAS. |  |  |  |
| 81 | (Waithaka et al., 2006) | Simulation integrated |  |  |  |  | The study integrated qualitative participatory approaches with mathematical programming and biophysical simulation modelling. A range of scenarios were assessed using the IMPACT tool to identify optimal farmer options that could improve household well-being. Food security was part of the discussion with farmers, but not directly a result of the modelling; however, outputs related to food security included yields, income, and food costs. |
| 82 | (Walker and Schulze, 2006) | Simulation biophysical | The CERES crop simulation model was used to simulate smallholder farmer production in response to different climate scenarios. Grain yield was the biophysical indicator used to imply **food availability** at the field level. A minimum yield target was specified as the "famine level". |  |  |  |  |
| 83 | (Wane et al., 2017) | Statistical |  | HFIAS data are converted to a "food security index" and a ordered probit model regresses four categories based on the FSI on Income mobility, income sources, number years supplying milk and number of livestock |  |  |  |
| 84 | (Whitney et al., 2017) | Simulation biophysical | This study used a probabilistic model to predict **food availability** measured as yields including both biophysical and social factors that affect cropping diversity and density and the subsequent yields. Yields of nutrients are implicitly assumed to be consumed. |  |  |  |  |
| 85 | (Wichern et al., 2017) | Other | **Food availability** is measured as average amount of food energy per adult male equivlent per day from own production and purchased.  The analysis assumes that all production not sold was available for household consumption, and that all other income was used to purchase maize. This measure appears to combine availability and access indicators. |  |  |  |  |
| 86 | (Wineman and Crawford, 2017) | Optimization | This study used an optimization approach to assess the effects of climate change on smallholder livelihoods, by allowing for adaptation strategies. Development and parameterization of the model involved various methods. Climate scenarios were simulated by changing crop yields. Simulated results included crop yields, available calories, and the likelihood of falling below a calorie threshold (an estimate of **food availability**). |  |  |  |  |
| 87 | (Winter et al., 2015) | Optimization | The village optimisation model used in the study included elements of multiple farms, resources, management, economic activities, and interaction with the market. The model is dynamic, and based on monthly decisions. One constraint was the income needed to meet energy and protein requirements. Food security is not explicity reported in the results section |  |  |  |  |
| 88 | (Wossen et al., 2018) | Simulation integrated | An agent-based model (MPMAS) was used to assess the effects of climate and price variability on household income and food security. **Food availability** is simulated through crop yields. | An agent-based model (MPMAS) was used to assess the effects of climate and price variability on household income and food security. **Food access** is simulated as household consumption based on consumption functions. |  |  |  |
| 89 | (Yiridoe et al., 2006) | Optimization | This study used linear programming to maximize income by allocating labour and capital. A minimum food requirement was used as a constraint on sales of crops. This was calculated based on average energy content of crops and average household energy requirements. Results are reported in terms of yields and income. |  |  |  |  |
| 90 | (Zereyesus et al., 2017) | Statistical |  | **Food access** measured by per capita food expenditures is regressed using 2SLS on household characteristics (type of work, age, schooling, credit, housing characteristics, household dependents), region and land productivity. |  |  |  |
| 91 | (Zheng et al., 2009) | Statistical | CART methods were used to determine the factors (abiotic, biotic or management) most responsible for soybean yield variability. Food security was discussed as a motivation for the study, but food security was not explicitly mentioned in the materials and methods or results. |  |  |  |  |
| 92 | (Asseng et al., 2019) | Simulation integrated | Using 32 dynamic models to predict the global wheat yield and protein under climate change scenarios |  |  |  |  |
| 93 | (Akter and Basher, 2014) | Statistical |  | **Food access** is measured with the FIES scale, then determinants are examined statistically. "Level 1" estimated probability of any food shortage observed with independent variables: Assets, per capita expenditure, area of cultivable land, religion, sex of houshold head, proportion food purchased, and geographic location.  "Level 2" estimated the probability of most severe food shortage in a given year (2007, 2008 or 2009) with independent variables: LN(remittance income in each of three years), LN(damage due to negative shocks in each of three years) and binary variable for whether experienced positive event during each of three years.  "Level 3" estimated the probabiilty of the most severe food shortage in a given year with independent variables price of rice, 'lean season' binary variables , and rice price interactions with type of farmer (self-employed, share cropper, non-agricultural day laborer, agricultural day laborer, fish/poultry/livestock, salaried employment). |  |  |  |
| 94 | (Antle et al., 2014) | equillibrium |  |  |  |  | The Trade-off Analysis Multi-Dimensional (TOA- MD) model simulates farmers’ choice between two production systems, the current system in use and a system with improved nutrient management practices involving increased fertilizer use, for given prices and costs of production. A market equilibrium model called TOA-ME that links site-specific process-based crop simulation models, econometric models of farm output supply and input demand, and a nutrient balance model—simulates the price- based trade-off curves and identifies the points of ME along those curves . The TOA-ME results are used to obtain equilibrium prices corresponding to the two technology scenarios. **Food availability** appears to be linked to food production (output supply), but is not specifically indicated. |
| 95 | (Bakker et al., 2018) | Economic | **Food availability** is determined by farmer decisions to cultivate land and sell harvested crops to storage. They are assumed to maximize profit with discounted futures by controlling crop area. Crop yields are determined with a simplifed version of DSSAT. Livestock managers control the number of livestock in the model regions. They sell meat and milk to storage, and they sell live animals to distribution as part of live animal trade in the model. Livestock managers make decisions about how many animals to trade or slaughter for meat (and hides), and they seek to maximize their utility subject to milk production and livestock reproduction rates as well as labour and operational costs. Their utility is a combination of profit plus the estimated value of the herd at the end of the 3-year horizon | **Food access** is measured as consumption, determined by household decisions to purchase food from storage, choosing the types and quantities of food to consume so as to maximize utility over a three-year time horizon. This utility is derived from an inverse demand function that incorporates dietary preferences, income and prices. Food consumption is disaggregated by age, gender, and per capita income level: income affects demand, whereas age and gender affect relative consumption in each income bracket. Nutrient intake is calculated based on average food composition and food consumption less estimated food losses. Labor productivity for working age males is multi-variate function of nutrients consumed. |  |  |  |
| 96 | (Chavez et al., 2015) | Simulation integrated | Hydrological and crop modelling and climate and weather modelling is used as input to produce grid-to-province PDFs of yield loss captured by weather indices. To model the vulnerability functions of crop yield to the combined or individual effects of precipitation variability and excess temperature exposure, generalized additive mixed models (GAMMs) are used. **Food availability** is represented only by yields, not production. |  |  |  |  |
| 97 | (Kun et al., 2015) | Optimization | Per capita grain production is used as an indicator of food security (better interpreted as a measure of **food availability**). Grain production is determined by an agent-based model that optimizes water allocation based on initial use requirements and alternative scenarios. |  |  |  |  |
| 98 | (Cordero-Ahiman. et al., 2017) | Statistical |  | **Food access** is measured with the FIES scale. A Binomal Logit model is estimated to assess the likelihood of food insecurity (yes or no) and an Ordered Logit Model to assess severity (one of four categories) using independent variables: income, consumed seed stocks prior to planting, maize production, whether household was engaged in temporary employment, age, household size, and whether they were beneficiaries of a social inclusion and conditional cash transfer program (Prospera). Odds ratios are calculated for the independent variables. |  |  |  |
| 99 | (Dermody et al., 2018) | Integrated simulation | Assessment of food security that integrates aspects of existing models and approaches in the fields of hydrology and integrated assessment modelling (IAM). The core of the framework is a multi-agent network of city agents connected by infrastructural trade networks. **Food availability** would be determined by food production, for which decisions are constrained by ecohydrological conditions. To capture ecohydrological conditions, we recommend tight coupling between a dynamic vegetation model (DGVM) such as the Lund–Potsdam–Jena managed Land (LPJ-ML) model and a complex hydrological model such as the PCRaster Global Water Balance (PCR-GLOBWB) model. | For **food access**, food consumption is the demand for each food commodity in a city and its hinterland, equal to (per capita demand taken from IAM × population). |  |  |  |
| 100 | (Sohoulande Djebou et al., 2017) | Statistical |  | **Food access** (insecurity) is measured with a non- standard experiential scale comprising five yes or no questions. A "food secure" household responded yes to fewer than 2 of the 5 questions. A multivariate logit model  estimated the impact of independent variables: land area farmed, use of irrigation, use of fertilizer and use of a tractor. Odds ratios are calculated for the independent variables. |  |  |  |
| 101 | (Dorosh et al., 2016) | Equilibrium | Four commodities are represented in a seven- equation partial equilibrium model: sorghum, maize, wheat, and rice. **Food availability** is modeled as production less losses plus imports which depends on relative prices. | **Food access** (consumption) is modeled as a log- linear function of per capita income and market prices. Aggregate income is calculated as the value of cereal production scaled up with a factor to account for downstream multiplier effects on processing, marketing, and consumption. |  |  |  |
| 102 | (Guillaume. et al., 2014) | Statistical | Linear conceptual model ("influence diagram", no empirical content) with an emphasis on the role of water availability. **Food availability** is shown as household-level own production. | **Food access** is shown as dependent on food availability from own production and other sources. The conceptual model is input into a logical process of how to model food security, with later emphasis on whether "food supply" equals "food demand". |  |  |  |
| 103 | (Haggblade et al., 2017) | Equilibrium | Five commodities are represented in a multi- market partial equilbrium model (sorghum/millet, rice, other starchy staple crops, high-value foods and non-foods), disaggregated by four household groups (rural poor, urban poor, rural non-poor and urban non- poor). **Food availability** is the sum of domestic production (assumed fixed) and imports (zero for sorghum/millet, fixed for high-value food imports, endogenous for other three products). | **Food access** is consumption, which equals estimated food demand, which is based on income and product prices using an Almost Ideal Demand System (AIDS) model. |  |  |  |
| 104 | (Harttgen et al., 2016) | Statistical |  | **Food access** (consumption) is specified at the household level as own production plus purchases, gifts or in-kind transfers for five main food groups (staples, pulses, vegetables and fruits, animal products and meal complements), expressed as caloric equivalents based on national household survey data. The impact of income shock on consumption is evaluated with a simple OLS regression of calories per day and per capita on log household income/expenditure. The effect of the food price shock is treated as an equivalent income shock, calculating the household-specific income equivalent of the price shock (i.e. maize quantity purchased ⁄ maize price increase), and then calculating how this income shock reduces calorie availability. |  |  |  |
| 105 | (Larson et al., 2014) | Others | **Food availability** is calculated as production and private carry-over of wheat, with planned production based on expected prices and input costs, and actual production differing from planned due to random exogenous shocks. | **Food access** is represented with a single demand equation for wheat with quantities as a function of price. Results focus on price variability and levels of strategic reserves; there is no reporting of food avaialbility or access outcomes. |  |  |  |
| 106 | (Lloyd et al., 2011) | Simulation integrated |  |  | A conceptual model indicates a single-equation relationship between degree of stunting (none/mild, moderate, severe) for children less than five years, and the independent variables Percent Undernourished, and a Development Score based on GDP per capita and its Gini coefficient, as well as an interaction term. The model is estimated by determining the 5th percentile value of ratio of proportion of children stunted and the Percent Undernourished across all countries and regions, and using that to estimate "food" causes and then attributing the remainder to "nonfood" causes. (Stunting is typically associated with **food utilization**.) |  |  |
| 107 | (Mainuddin et al., 2011) | Simulation integrated | The crop simulation model *AquaCrop* was used to predict rice yields in 14 zones of the study region. **Food availability** is represented only by yields, not production. |  |  |  |  |
| 108 | (Asseng et al., 2018) | Simulation integrated | DSSAT models used to predict wheat yield and food gap (self-sufficiency) in Egypt under climate change, population, technology, and adaptation scenarios |  |  |  |  |
| 109 | (Mason-D'Croz et al., 2016) | Simulation integrated | Analyses use the GLOBIOM and IMPACT models, which are global partial equilibrium models with detailed representation of the agricultural sector. GLOBIOM represents 18 crops, 7 livestock products, 5 forest products, and 9 bioenegy products for 30 country-based regions, with linked regional markets. **Food availability** is determined by crop yields and land area, where the yields are determined by the EPIC crop model. | **Food access** (consumption) is estimated using double-log demand systems based on price and income to model consumer food demand, considering both a dynamic adjustment to demand based on income growth as well as a demand response based on prices. Additional description of the IMPACT model is provided below in the discussion of Springmann et al (2016). |  |  |  |
| 110 | (Montella et al., 2015) | Simulation integrated |  |  |  |  | This is an IT module linking different models and data sources, including climate and crops (e.g., DSSAT and APSIM). Examples shown include temperature and crop yields, but no more specific food availability or access metrics. |
| 111 | (Moore et al., 2012) | Simulation integrated | This is a series of linked land-use, climate and crop simulation models. The spatial distribution of maize yields and distribution of maize yield changes are the principal outputs, but no specific food availability or access metrics. |  |  |  |  |
| 112 | (Oehmke et al., 2018) | Statistical |  |  |  |  | This analysis is based on a three-person prisoner's dilemmma game simulated 10,000 times for a 500-year period to assess stylized payoff outcomes. There is no direct representation of food security metrics in the analysis or the discussion. |
| 113 | (Paeth et al., 2008) | Statistical | Uses a stepwise multiple linear regression analysis (Model output statistics, MOS) approach to relate yields for 8 crops (peanuts, cotton, beans, yams, maize, manioc, rice and sorghum) in 7 regions to climate variables (rainfall, temperature, humidity) from the regional climate model REMO. Crop yield changes are the principal outputs, but no specific food availability or access metrics |  |  |  |  |
| 114 | (Palazzo et al., 2017) | Simulation integrated | Analyses use the GLOBIOM and IMPACT models, which are global partial equilibrium models with detailed representation of the agricultural sector. GLOBIOM represents 18 crops, 7 livestock products, 5 forest products, and 9 bioenegy products for 30 country-based regions, with linked regional markets. **Food availability** is determined by crop yields and land area, where the yields are determined by the EPIC crop model. | **Food access** (consumption) is estimated using double-log demand systems based on price and income to model consumer food demand, considering both a dynamic adjustment to demand based on income growth as well as a demand response based on prices. Additional description of the IMPACT model is provided below in the discussion of Springmann et al (2016). |  |  |  |
| 115 | (Springmann et al., 2016) | Integrated simulation | The analysis uses the IMPACT model, a multi- region partial equilibrium model that uses economic, water, and crop models to simulate global food production, consumption and trade of 62 agricultural commodities for 159 world regions. National **food availability** is calculated based on crop yields and land areas. Crop yields are a function of commodity prices, prices of inputs, available water, climate, and exogenous trend factors. Crop area is specified as an area demand function with respect to changes in the marginal revenue product, changes in land cost, and exogenous nonprice trends in harvested area. | **Food access** (consumption) is specified as food demand less estimated losses. Food demand is a function of the price of the commodity and the prices of other competing commodities, per capita income, and total population. Per capita income and population increase annually according to country-specific population and income growth rates. |  |  |  |
| 116 | (Tabeau et al., 2017) | CGE | The analysis uses a global computable general equilibrium model (CGE) with 10 subregions. **Food availabiility** is based on production, where producers are price takers that choose the cheapest combination of imperfectly substitutable labour, capital, land, natural resources and intermediates. Production relationships use a multilevel sector specific nested constant elasticity of substitution (CES) production function, allowing for substitution between primary (land, labour, capital and natural resources) and intermediate production factors, and substitution between different intermediate input components e.g. energy sources, and animal feed components. | Households are assumed to allocate income to savings and consumption expenditures according to fixed budget shares following a Cobb-Douglas (CD) expenditure function. **Food access** (consumption) is determined by the allocation of private consumption expenditures across commodities through a statistically estimated demand system. |  |  |  |
| 117 | (Wailes et al., 2015) | Simulation integrated | National-level **food availability** is represented with price-responsive rice supply functions and import functions. The analysis uses two models, Arkansas Global Rice Model (AGRM, a stuctural econometric model) and RiceFlow (a spatial partial equilibrium model) to estimate impacts on production and consumption of national rice development strategies. | Aggregate food consumption (**food access**) is represented with price-responsive demand functions that allow for imperfect substitution between domestic and imported rice. |  |  |  |
| 118 | (Wu et al., 2016) |  |  |  |  |  | "Food security" is proxied with ten grain production-consumption coordination indexes, which include production-consumption, price fluctuation, production fluctuation, consumption fluctuation, degree of dependence on foreign trade, etc., apparently assuming that self- sufficiency is an indicator of food security. The analysis does not report any direct metrics of food security, only intertemporal values of three calculated coordination indexes. |
| 119 | (Kheir et al., 2019) | Simulation integrated | Determining wheat yield and gap of production using Multi-model ensemble |  |  |  |  |
| 120 | (Kheir et al., 2021) | Optimization | Optimizing wheat yield and WUE using DSSAT models |  |  |  |  |
| 121 | (Moore et al., 2014) | Integrated simulation |  | Rule templates make it easier to reuse complex management scripts by combining commonly used collections of rules with an interface that allows a simulation builder to input key parameters. |  |  |  |
| 122 | (Fujimori et al., 2019) | Integrated simulation | Using a multi-model comparison exercise, trade-offs between food security and climate mitigation were investigated. |  |  |  |  |

**References:**

Adewumi, M.O., Animashaun, J.O., 2013. Households’ dietary diversity, farm income and technical efficiency correlates: Empirical evidence from small-scale farming households in Nigeria AGRIS on-line Papers in Economics and Informatics 5(4), 3-11.

Ahmad, M., Mustafa, G., Iqbal, M., 2016. Impact of farm households' adaptations to climate change on food security: Evidence from different agro-ecologies of Pakistan. Pakistan Development Review, 55(4), 561-588. DOI: 510.30541/v30555i30544I-IIpp.30561-30588.

Akerele, D., Sanusi, R.A., Fadare, O.A., Ashaolu, O.F., 2017. Factors Influencing Nutritional Adequacy among Rural Households in Nigeria: How Does Dietary Diversity Stand among Influencers? . Ecology of Food and Nutrition, 56(2), 187-203. doi: 110.1080/03670244.03672017.01281127.

Akerele, D., Shittu, A.M., 2017. Can food production diversity influence farm households’ dietary diversity? An appraisal from two-dimensional food diversity measures. International Journal of Social Economics.

Akinola, A.A., Alene, A.D., Adeyemo, R., Sanogo, D., Olanrewaju, A.S., Nwoke, C., Nziguheba, G., 2010. Determinants of adoption and intensity of use of balance nutrient management systems technologies in the northern Guinea savanna of Nigeria. Quarterly Journal of International Agriculture 49(892-2016-65208), 25-45.

Akter, S., Basher, S.A., 2014. The impacts of food price and income shocks on household food security and economic well-being: Evidence from rural Bangladesh. Global Environmental Change 25, 150-162.

Ali, A., Erenstein, O., 2017. Assessing farmer use of climate change adaptation practices and impacts on food security and poverty in Pakistan. Climate Risk Management 16, 183-194.

Alwang, J., Siegel, P.B., 1999. Labor Shortages on Small Landholdings in Malawi: Implications for Policy Reforms. World Development 27(8), 1461-1475.

Amede, T., Delve, R.J., 2008. MODELLING CROP–LIVESTOCK SYSTEMS FOR ACHIEVING FOOD SECURITY AND INCREASING PRODUCTION EFFICIENCIES IN THE ETHIOPIAN HIGHLANDS. Experimental Agriculture 44(4), 441-452.

Antle, J.M., Stoorvogel, J.J., Valdivia, R.O., 2014. New parsimonious simulation methods and tools to assess future food and environmental security of farm populations. Philosophical Transactions of the Royal Society B: Biological Sciences 369(1639), 20120280.

Asseng, S., Kheir, A.M.S., Kassie, B.T., Hoogenboom, G., Abdelaal, A.I.N., Haman, D.Z., Ruane, A.C., 2018. Can Egypt become self-sufficient in wheat? Environmental Research Letters 13(9), 094012.

Asseng, S., Martre, P., Maiorano, A., Rötter, R.P., O’Leary, G.J., Fitzgerald, G.J., Girousse, C., Motzo, R., Giunta, F., Babar, M.A., Reynolds, M.P., Kheir, A.M.S., Thorburn, P.J., Waha, K., Ruane, A.C., Aggarwal, P.K., Ahmed, M., Balkovič, J., Basso, B., Biernath, C., Bindi, M., Cammarano, D., Challinor, A.J., De Sanctis, G., Dumont, B., Eyshi Rezaei, E., Fereres, E., Ferrise, R., Garcia-Vila, M., Gayler, S., Gao, Y., Horan, H., Hoogenboom, G., Izaurralde, R.C., Jabloun, M., Jones, C.D., Kassie, B.T., Kersebaum, K.-C., Klein, C., Koehler, A.-K., Liu, B., Minoli, S., Montesino San Martin, M., Müller, C., Naresh Kumar, S., Nendel, C., Olesen, J.E., Palosuo, T., Porter, J.R., Priesack, E., Ripoche, D., Semenov, M.A., Stöckle, C., Stratonovitch, P., Streck, T., Supit, I., Tao, F., Van der Velde, M., Wallach, D., Wang, E., Webber, H., Wolf, J., Xiao, L., Zhang, Z., Zhao, Z., Zhu, Y., Ewert, F., 2019. Climate change impact and adaptation for wheat protein. Global Change Biology 25(1), 155-173.

Azeem, M.M., Mugera, A.W., Schilizzi, S., 2016. Living on the edge: Household vulnerability to food-insecurity in the Punjab, Pakistan. Food Policy 64, 1-13.

Bacon, C.M., Sundstrom, W.A., Flores Gómez, M.E., Ernesto Méndez, V., Santos, R., Goldoftas, B., Dougherty, I., 2014. Explaining the ‘hungry farmer paradox’: Smallholders and fair trade cooperatives navigate seasonality and change in Nicaragua's corn and coffee markets. Global Environmental Change 25, 133-149.

Bakker, C., Zaitchik, B.F., Siddiqui, S., Hobbs, B.F., Broaddus, E., Neff, R.A., Haskett, J., Parker, C.L., 2018. Shocks, seasonality, and disaggregation: Modelling food security through the integration of agricultural, transportation, and economic systems. Agricultural Systems 164, 165-184.

Baran, E., Jantunen, T., Chheng, P., Hoanh, C.T., 2010. Integrated management of aquatic resources: a Bayesian approach to water control and trade-offs in Southern Vietnam. Tropical deltas and coastal zones: food production, communities and environment at the land-water interface, 133-142.

Bashir, M.K., Schilizzi, S., Mohammad, S., 2014. Do demand side policies improve the food security of landless rural households? Investigating Pakistan’s achievements in the Punjab. Journal of Animal and Plant Sciences, 24(5), 1554-1564.

Beghin, J.C., Teshome, Y., 2017. The Coffee-Food Security Interface for Subsistence Households in Jimma Zone Ethiopia, World Agricultural Resources and Food Security. Emerald Publishing Limited, pp. 221-240.

Beyene, F., Muche, M., 2010. Determinants of food security among rural households of central Ethiopia: an empirical analysis Quarterly Journal of International Agriculture 49(4), 299-318.

Beyene, L.M., Engida, E., 2016. Public investment in irrigation and training, growth and poverty reduction in Ethiopia. International Journal of Microsimulation 9(1), 86-108.

Bharwani, S., Bithell, M., Downing, T.E., New, M., Washington, R., Ziervogel, G., 2005. Multi-agent modelling of climate outlooks and food security on a community garden scheme in Limpopo, South Africa. Philosophical transactions of the Royal Society of London. Series B, Biological sciences 360(1463), 2183-2194.

Chavez, E., Conway, G., Ghil, M., Sadler, M., 2015. An end-to-end assessment of extreme weather impacts on food security. Nature Climate Change 5(11), 997-1001.

Cordero-Ahiman., Otilia-Vanessa., Santellano-Estrada, E., Garrido, A., 2017. Explaining food insecurity among indigenous households of the Sierra Tarahumara in the Mexican state of Chihuahua. Spanish Journal of Agricultural Research 15(1), e0106.

Darsonon., 2017. Staple Food Self-Sufficiency of Farmers Household Level in the Great Solo. . IOP Conference Series: Materials Science and Engineering, 193(1).

Dermody, B.J., Sivapalan, M., Stehfest, E., van Vuuren, D.P., Wassen, M.J., Bierkens, M.F.P., Dekker, S.C., 2018. A framework for modelling the complexities of food and water security under globalisation. Earth Syst. Dynam. 9(1), 103-118.

Dhakal, B., Bigsby, H., Cullen, R., 2010. Forests for Food Security and Livelihood Sustainability: Policy Problems and Opportunities for Small Farmers in Nepal. Journal of Sustainable Agriculture 35(1), 86-115.

Di Falco, S., Veronesi, M., Yesuf, M., 2011. Does Adaptation to Climate Change Provide Food Security? A Micro-Perspective from Ethiopia. American Journal of Agricultural Economics 93(3), 829-846.

Dil Farzana, F., Rahman, A.S., Sultana, S., Raihan, M.J., Haque, M.A., Waid, J.L., Choudhury, N., Ahmed, T., 2017. Coping strategies related to food insecurity at the household level in Bangladesh. PLoS ONE (April 14, 2017), https://doi.org/10.1371/journal.pone.0171411.

Djanibekov, U., Djanibekov, N., Khamzina, A., Bhaduri, A., Lamers, J.P.A., Berg, E., 2013. Impacts of innovative forestry land use on rural livelihood in a bimodal agricultural system in irrigated drylands. Land Use Policy 35, 95-106.

Dobbie., Samantha., Balbi, S., 2017. Design of an Empirical Agent-Based Model to Explore Rural Household Food Security Within a Developing Country Context, pp. . 81–94 in Advances in Social Simulation 2015, Wander Jager, Rineke Verbrugge, Andreas Flache, Gert de Roo, Lex Hoogduin, Charlotte Hemelrijk, Ed. Springer International Publishing AG 2017 [Advances in Intelligent Systems and Computing 528]. .

Dorosh, P.A., Rashid, S., van Asselt, J., 2016. Enhancing food security in South Sudan: the role of markets and regional trade. Agricultural Economics 47(6), 697-707.

Ferdous, Z., Datta, A., Anal, A.K., Anwar, M., Khan, A.S.M.M.R., 2016. Development of home garden model for year round production and consumption for improving resource-poor household food security in Bangladesh. NJAS: Wageningen Journal of Life Sciences 78(1), 103-110.

Fujimori, S., Hasegawa, T., Krey, V., Riahi, K., Bertram, C., Bodirsky, B.L., Bosetti, V., Callen, J., Després, J., Doelman, J., Drouet, L., Emmerling, J., Frank, S., Fricko, O., Havlik, P., Humpenöder, F., Koopman, J.F.L., van Meijl, H., Ochi, Y., Popp, A., Schmitz, A., Takahashi, K., van Vuuren, D., 2019. A multi-model assessment of food security implications of climate change mitigation. Nature Sustainability 2(5), 386-396.

Gangwar, B., Subash, N., Ravisankar, N., 2016. Farming system approach to meet the challenges from extreme weather. Mausam 67(1), 15-26.

Guillaume., Joseph, H.A., Kummu, M., Miina, P., Olli, V., 2014. A conceptual model to guide exploration of global food-water security. International Congress on Environmental Modelling and Software 20.

Habyarimana, J.B., Nkunzimana, T., 2017. Policy Reforms and Rural Livelihoods Sustainability: Challenges and Opportunities — Empirical Evidence from the Adoption of the Land Use Consolidation (LUC) Policy in Rwanda. African Development Review 29(S2), 96-108.

Hadush, M., 2017. Implication of animal feed and water scarcity on labor allocation, food production and per capita food consumption in Tigrai region, Ethiopia. Journal of Economic Development, 42(4), 59-93.

Haggblade, S., Me-Nsope, N.M., Staatz, J.M., 2017. Food security implications of staple food substitution in Sahelian West Africa. Food Policy 71, 27-38.

Hammond, J., Fraval, S., Etten, J., Suchini, J.G., Mercado, L., Pagella, T., Frelat, R., Lannerstad, M., Douxchamps, S., Teufel, N., Valbuena, D., Wijk, M.T., 2018. The Rural Household Multi-Indicator Survey (RHoMIS) for rapid characterisation of households to inform climate smart agriculture interventions: Description and applications in East Africa and Central America. Archaeology Agricultural Systems 151:225–233.

Hartter, J., Boston, K., 2007. An integrated approach to modeling resource utilization for rural communities in developing countries. Journal of Environmental Management 85(1), 78-92.

Harttgen, K., Klasen, S., Rischke, R., 2016. Analyzing nutritional impacts of price and income related shocks in Malawi: Simulating household entitlements to food. Food Policy 60, 31-43.

Hoddinott, J., Berhane, G., Gilligan, D.O., Kumar, N., Seyoum Taffesse, A., 2012. The Impact of Ethiopia's Productive Safety Net Programme and Related Transfers on Agricultural Productivity. Journal of African Economies 21(5), 761-786.

Holden, S., Shiferaw, B., 2004. Land degradation, drought and food security in a less-favoured area in the Ethiopian highlands: a bio-economic model with market imperfections. Agricultural Economics 30(1), 31-49.

Holden, S.T., Bekele, S., Shiferaw, B.A., Pender, J., 2005. Policy analysis for sustainable land management and food security in Ethiopia: A bioeconomic model with market imperfections. Intl Food Policy Res Inst.

Hussien, W.e.A., Memon, F.A., Savic, D.A., 2017. An integrated model to evaluate water-energy-food nexus at a household scale. Environmental Modelling & Software 93, 366-380.

Ibrahim, H., Bello, M., Ibrahim, H., 2009. Food security and resource allocation among farming households in north central Nigeria. Pakistan Journal of Nutrition 8(8), 1235-1239.

Inder, B., Kabore, C., Nolan, S., Cornwell, K., Contreras Suarez, D., Crawford, A., Kamara, J.K., 2017. Livelihoods and Child Welfare among Poor Rural Farmers in East Africa. African Development Review 29(2), 169-183.

Islam, A.H.M.S., von Braun, J., Thorne-Lyman, A.L., Ahmed, A.U., 2018. Farm diversification and food and nutrition security in Bangladesh: empirical evidence from nationally representative household panel data. Food Security 10(3), 701-720.

Jiang, Q., Deng, X.Z., Yan, H.M., Liu, D.D., Qu, R.J., 2012. Identification of food security in the mountainous guyuan prefecture of China by exploring changes of food production.

Joshi, G.R., Joshi, B., 2017. Household food security: Trends and determinants in mountainous districts of Nepal.

Kaminski, J., Thomas, A., 2011. Land use, production growth, and the institutional environment of smallholders: evidence from Burkinabè cotton farmers. Land Economics, 87(1), 161-182.

Karki, T.B., Sah, S.K., Thapa, R.B., McDonald, A.J., Davis, A.S., 2015. Identifying pathways for improving household food self-sufficiency outcomes in the hills of Nepal. PLoS ONE, June 5, 2015https://doi.org/10.1371/journal.pone.01275 13.

Kassie, M., Pender, J., Yesuf, M., Kohlin, G., Bluffstone, R., Mulugeta, E., 2008. Estimating returns to soil conservation adoption in the northern Ethiopian highlands. Agricultural Economics 38(2), 213-232.

Kassie, M., Stage, J., Teklewold, H., Erenstein, O., 2015. Gendered food security in rural Malawi: why is women’s food security status lower? Food Security 7(6), 1299-1320.

Kheir, A.M.S., Alrajhi, A.A., Ghoneim, A.M., Ali, E.F., Magrashi, A., Zoghdan, M.G., Abdelkhalik, S.A.M., Fahmy, A.E., Elnashar, A., 2021. Modeling deficit irrigation-based evapotranspiration optimizes wheat yield and water productivity in arid regions. Agricultural Water Management 256, 107122.

Kheir, A.M.S., El Baroudy, A., Aiad, M.A., Zoghdan, M.G., Abd El-Aziz, M.A., Ali, M.G.M., Fullen, M.A., 2019. Impacts of rising temperature, carbon dioxide concentration and sea level on wheat production in North Nile delta. Science of The Total Environment 651, 3161-3173.

Kokoye, S.E.H., Tovignan, S.D., Yegbemey, R.N., 2013. Land Use Change and Food Security: Has Introduction of Rice Production in Cotton Zone in Benin Met Optimal Allocation of Resources by Households?. In: Behnassi M., Pollmann O., Kissinger G. (eds) Sustainable Food Security in the Era of Local and Global Environmental Change. Springer, Dordrecht https://doi.org/10.1007/978-94-007-6719-5_18.

Kowero, G., Nhantumbo, I., Tchale, H., 2005. Reconciling household goals in southern African woodlands using weighted goal programming. International Forestry Review 7(4), 294-304. doi: 210.1505/ifor.2005.1507.1504.1294.

Kun, C., Fu, Q., Li, T., Jiang, Q., Liu, W., 2015. Regional food security risk assessment under the coordinated development of water resources. Natural Hazards 78, 603–619.

Laborte, A.G., Schipper, R.A., Van Ittersum, M.K., Van Den Berg, M.M., Van Keulen, H., Prins, A.G., Hossain, M., 2009. Farmers' welfare, food production and the environment: a model-based assessment of the effects of new technologies in the northern Philippines. NJAS - Wageningen Journal of Life Sciences 56(4), 345-373.

Laborte, A.G., Van Ittersum, M.K., Van den Berg, M.M., 2007. Multi-scale analysis of agricultural development: A modelling approach for Ilocos Norte, Philippines. Agricultural Systems 94(3), 862-873.

Larson, D.F., Lampietti, J., Gouel, C., Cafiero, C., Roberts, J., 2014. Food Security and Storage in the Middle East and North Africa. The World Bank Economic Review 28(1), 48-73.

Lázár, A.N., Clarke, D., Adams, H., Akanda, A.R., Szabo, S., Nicholls, R.J., Matthews, Z., Begum, D., Saleh, A.F.M., Abedin, M.A., Payo, A., Streatfield, P.K., Hutton, C., Mondal, M.S., Moslehuddin, A.Z.M., 2015. Agricultural livelihoods in coastal Bangladesh under climate and environmental change – a model framework. Environmental Science: Processes & Impacts 17(6), 1018-1031.

Leonardo, W., van de Ven, G.W.J., Kanellopoulos, A., Giller, K.E., 2018. Can farming provide a way out of poverty for smallholder farmers in central Mozambique? Agricultural Systems 165, 240-251.

Lloyd, S.J., Kovats, R.S., Chalabi, Z., 2011. Climate change, crop yields, and undernutrition: development of a model to quantify the impact of climate scenarios on child undernutrition. Environmental health perspectives 119(12), 1817-1823.

Louhichi, K., Gomez y Paloma, S., 2014. A farm household model for agri-food policy analysis in developing countries: Application to smallholder farmers in Sierra Leone. Food Policy 45, 1-13.

Maatman, A., Sawadogo, H., Schweigman, C., Ouedraogo, A., 1998. Application of zai and rock bunds in the northwest region of Burkina Faso: Study of its impact on household level by using a stochastic linear programming model. Netherlands Journal of Agricultural Science 46(1), 123-136.

Magcale-Macandog, D.B., Rañola, F.M., Rañola, R.F., Ani, P.A.B., Vidal, N.B., 2010. Enhancing the food security of upland farming households through agroforestry in Claveria, Misamis Oriental, Philippines. Agroforestry Systems 79(3), 327-342.

Mainuddin, M., Kirby, M., Hoanh, C.T., 2011. Adaptation to climate change for food security in the lower Mekong Basin. Food Security 3(4), 433-450.

Marsh, T.L., Yoder, J., Deboch, T., McElwain, T.F., Palmer, G.H., 2016. Livestock vaccinations translate into increased human capital and school attendance by girls. Science advances 2(12), e1601410.

Mason-D'Croz, D., Vervoort, J., Palazzo, A., Islam, S., Lord, S., Helfgott, A., Havlík, P., Peou, R., Sassen, M., Veeger, M., van Soesbergen, A., Arnell, A.P., Stuch, B., Arslan, A., Lipper, L., 2016. Multi-factor, multi-state, multi-model scenarios: Exploring food and climate futures for Southeast Asia. Environmental Modelling & Software 83, 255-270.

Modi, A.T., 2015. A simple model to evaluate integrated vegetable production for food security in KwaZulu-Natal, South Africa. Food Research International 76, 946-952.

Molua, E.L., 2012. Gendered response and risk‐coping capacity to climate variability for sustained food security in Northern Cameroon. International Journal of Climate Change Strategies and Management 4(3), 277-307.

Montella, R., Kelly, D., Xiong, W., Brizius, A., Elliott, J., Madduri, R., Maheshwari, K., Porter, C., Vilter, P., Wilde, M., Zhang, M., Foster, I., 2015. FACE-IT: A science gateway for food security research. Concurrency and Computation: Practice and Experience 27(16), 4423-4436.

Moore, A.D., Holzworth, D.P., Herrmann, N.I., Brown, H.E., de Voil, P.G., Snow, V.O., Zurcher, E.J., Huth, N.I., 2014. Modelling the manager: Representing rule-based management in farming systems simulation models. Environmental Modelling & Software 62, 399-410.

Moore, N., Alagarswamy, G., Pijanowski, B., Thornton, P., Lofgren, B., Olson, J., Andresen, J., Yanda, P., Qi, J., 2012. East African food security as influenced by future climate change and land use change at local to regional scales. Climatic Change 110(3), 823-844.

Murungweni, C., van Wijk, M.T., Andersson, J.A., Smaling, E.M.A., Giller, K.E., 2011. Application of Fuzzy Cognitive Mapping in Livelihood Vulnerability Analysis. Ecology and Society 16(4).

N’Danikou, S., Vodouhe, R.S., Bellon, M.R., Sidibé, A., Coulibaly, H., 2017. Foraging Is Determinant to Improve Smallholders’ Food Security in Rural Areas in Mali, West Africa. Sustainability 9(11).

Niragira, S., D'Haese, M., D'Haese, L., Ndimubandi, J., Desiere, S., Buysse, J., 2015. Food for survival: Diagnosing crop patterns to secure lower threshold food security levels in farm households of Burundi. . Food and Nutrition Bulletin 36(2), 196-210. doi: 110.1177/0379572115587491.

Nkegbe, P.K., Abu, B.M., Issahaku, H., 2017. Food security in the Savannah Accelerated Development Authority Zone of Ghana: an ordered probit with household hunger scale approach. Agriculture & Food Security 6(1), 35.

Nyaga, E.K., Doppler, W., 2009. Combining principal component analysis and logistic regression models to assess household level food security among smallholder cash crop producers in Kenya. Quarterly Journal of international agriculture 48(1), 5-23.

Obayelu, O.A., Onasanya, O.A., 2016. Maize biodiversity and food security status of rural households in the derived Guinea savannah of Oyo state, Nigeria. Agriculturae Conspectus Scientificus 81(4), 241-250.

Oehmke, J.F., Young, S.L., Bahiigwa, G., Keizire, B.B., Post, L.A., 2018. The Behavioral-Economics Basis of Mutual Accountability to Achieve Food Security. Politics & Policy 46(1), 32-57.

Ogot, N.O., Pittchar, J.O., Midega, C.A.O., Khan, Z.R., 2017. Impact of push-pull technology on the nutritional status of farmers' children in Western Kenya. African Journal of Food, Agriculture, Nutrition and Development 17(4), 12953-12974.

Paeth, H., Capo-Chichi, A., Endlicher, W., 2008. Climate Change and Food Security in Tropical West Africa — A Dynamic-Statistical Modelling Approach. Erdkunde 62(2), 101-115.

Palazzo, A., Vervoort, J.M., Mason-D’Croz, D., Rutting, L., Havlík, P., Islam, S., Bayala, J., Valin, H., Kadi Kadi, H.A., Thornton, P., Zougmore, R., 2017. Linking regional stakeholder scenarios and shared socioeconomic pathways: Quantified West African food and climate futures in a global context. Global Environmental Change 45, 227-242.

Radchenko, N., Corral, P., 2018. Agricultural Commercialisation and Food Security in Rural Economies: Malawian Experience. The Journal of Development Studies 54(2), 256-270.

Rader, M., Kirshen, P., Roncoli, C., Hoogenboom, G., Ouattara, F., 2009. Agricultural Risk Decision Support System for Resource-Poor Farmers in Burkina Faso, West Africa. Journal of Water Resources Planning and Management 135(5), 323-333.

Ragasa, C., Mazunda, J., 2018. The impact of agricultural extension services in the context of a heavily subsidized input system: The case of Malawi. World Development 105, 25-47.

Reincke, K., Vilvert, E., Fasse, A., Graef, F., Sieber, S., Lana, M.A., 2018. Key factors influencing food security of smallholder farmers in Tanzania and the role of cassava as a strategic crop. Food Security 10(4), 911-924.

Rigolot, C., de Voil, P., Douxchamps, S., Prestwidge, D., Van Wijk, M., Thornton, P.K., Rodriguez, D., Henderson, B., Medina, D., Herrero, M., 2017. Interactions between intervention packages, climatic risk, climate change and food security in mixed crop–livestock systems in Burkina Faso. Agricultural Systems 151, 217-224.

Salazar, L., Aramburu, J., González-Flores, M., Winters, P., 2016. Sowing for food security: A case study of smallholder farmers in Bolivia. Food Policy 65, 32-52.

Sassi, M., Cardaci, A., 2013. Impact of rainfall pattern on cereal market and food security in Sudan: Stochastic approach and CGE model. Food Policy 43, 321-331.

Seaman, J.A., Sawdon, G.E., Acidri, J., Petty, C., 2014. The Household Economy Approach. Managing the impact of climate change on poverty and food security in developing countries. Climate Risk Management 4-5, 59-68.

Sibhatu, K.T., Krishna, V.V., Qaim, M., 2015. Production diversity and dietary diversity in smallholder farm households. PNAS.

Sohoulande Djebou, D.C., Price, E., Kibriya, S., Ahn, J., 2017. Comparative Analysis of Agricultural Assets, Incomes and Food Security of Rural Households in Ghana, Senegal and Liberia. Agriculture 7(5).

Springmann, M., Mason-D’Croz, D., Robinson, S., Garnett, T., Godfray, C.J., Gollin, D., Rayner, M., Ballon, P., Scarborough, P., 2016. Global and regional health effects of future food production under climate change: a modelling study. Lancet 387, 1937–1946.

Stephens, E.C., Nicholson, C.F., Brown, D.R., Parsons, D., Barrett, C.B., Lehmann, J., Mbugua, D., Ngoze, S., Pell, A.N., Riha, S.J., 2012. “Modeling the impact of natural resource-based poverty traps on food security in Kenya: The Crops, Livestock and Soils in Smallholder Economic Systems (CLASSES) model”. Food Security 4(3), 423-439.

Suneetha, P., Yirgu, T., 2010. An agro-ecological assessment of household food security in Basso catchment, Ethiopia. Transactions of the Institute of Indian Geographers.

Szabo, S., Hossain, M.S., Adger, W.N., Matthews, Z., Ahmed, S., Lázár, A.N., Ahmad, S., 2016. Soil salinity, household wealth and food insecurity in tropical deltas: evidence from south-west coast of Bangladesh. Sustainability Science 11(3), 411-421.

Tabeau, A., van Meijl, H., Overmars, K.P., Stehfest, E., 2017. REDD policy impacts on the agri-food sector and food security. Food Policy 66, 73-87.

Tesfaye, A., Bogale, A., Namara, R.E., Bacha, D., 2008. The impact of small-scale irrigation on household food security: The case of Filtino and Godino irrigation schemes in Ethiopia. Irrigation and Drainage Systems 22(2), 145-158.

Thorlakson, T., Neufeldt, H., 2012. Reducing subsistence farmers’ vulnerability to climate change: evaluating the potential contributions of agroforestry in western Kenya. Agriculture & Food Security 1(1), 15.

Thornton, P.K., BurnSilver, S.B., Boone, R.B., Galvin, K.A., 2006. Modelling the impacts of group ranch subdivision on agro-pastoral households in Kajiado, Kenya. Agricultural Systems 87(3), 331-356.

Tingem, M., Rivington, M., Colls, J., 2008. Climate variability and maize production in Cameroon: Simulating the effects of extreme dry and wet years. Singapore Journal of Tropical Geography 29(3), 357-370.

Tittonell, P., van Wijk, M.T., Herrero, M., Rufino, M.C., de Ridder, N., Giller, K.E., 2009. Beyond resource constraints – Exploring the biophysical feasibility of options for the intensification of smallholder crop-livestock systems in Vihiga district, Kenya. Agricultural Systems 101(1), 1-19.

Traore, B., Descheemaeker, K., van Wijk, M.T., Corbeels, M., Supit, I., Giller, K.E., 2017. Modelling cereal crops to assess future climate risk for family food self-sufficiency in southern Mali. Field Crops Research 201, 133-145.

Traoré, S.A., Reiber, C., Megersa, B., Zárate, A.V., 2018. Contribution of cattle of different breeds to household food security in southern Mali. Food Security 10(3), 549-560.

Wailes, E.J., Durand-Morat, A., Diagne, M., 2015. Regional and National Rice Development Strategies for Food Security in West Africa, Food Security in an Uncertain World. Emerald Publishing Ltd, pp. 255-268.

Waithaka, M.M., Thornton, P.K., Herrero, M., Shepherd, K.D., 2006. Bio-economic evaluation of farmers’ perceptions of viable farms in western Kenya. Agricultural Systems 90(1), 243-271.

Walker, N.J., Schulze, R.E., 2006. An assessment of sustainable maize production under different management and climate scenarios for smallholder agro-ecosystems in KwaZulu-Natal, South Africa. Physics and Chemistry of the Earth, Parts A/B/C 31(15), 995-1002.

Wane, A., Cadihon, J.J., Yauck, M., 2017. Socioeconomic impacts of innovative dairy supply chain practices--The case of the Laiterie du Berger in Senegalese Sahel. International Food and Agribusiness Management Review, 20, DOI: 10.22434/IFAMR22015.20218.

Whitney, C.W., Tabuti, J.R.S., Hensel, O., Yeh, C.-H., Gebauer, J., Luedeling, E., 2017. Homegardens and the future of food and nutrition security in southwest Uganda. Agricultural Systems 154, 133-144.

Wichern, J., van Wijk, M.T., Descheemaeker, K., Frelat, R., van Asten, P.J.A., Giller, K.E., 2017. Food availability and livelihood strategies among rural households across Uganda. Food Security 9(6), 1385-1403.

Wineman, A., Crawford, E.W., 2017. Climate change and crop choice in Zambia: A mathematical programming approach. NJAS: Wageningen Journal of Life Sciences 81(1), 19-31.

Winter, E., Faße, A., Frohberg, K., 2015. Food security, energy equity, and the global commons: a computable village model applied to sub-Saharan Africa. Regional Environmental Change 15(7), 1215-1227.

Wossen, T., Berger, T., Haile, M.G., Troost, C., 2018. Impacts of climate variability and food price volatility on household income and food security of farm households in East and West Africa. Agricultural Systems 163, 7-15.

Wu, J., Zhang, J., Wang, S., Kong, F., 2016. Assessment of Food Security in China: A New Perspective Based on Production-Consumption Coordination. Sustainability 8(3).

Yiridoe, E.K., Langyintuo, A.S., Dogbe, W., 2006. Economics of the impact of alternative rice cropping systems on subsistence farming: Whole-farm analysis in northern Ghana. Agricultural Systems 91(1), 102-121.

Zereyesus, Y.A., Embaye, W.T., Tsiboe, F., Amanor-Boadu, V., 2017. Implications of Non-Farm Work to Vulnerability to Food Poverty-Recent Evidence From Northern Ghana. World Development 91, 113-124.

Zheng, H., Chen, L., Han, X., Zhao, X., Ma, Y., 2009. Classification and regression tree (CART) for analysis of soybean yield variability among fields in Northeast China: The importance of phosphorus application rates under drought conditions. Agriculture, Ecosystems & Environment 132(1), 98-105.
